# Genotyping of *Bacillus anthracis *strains based on automated capillary 25-loci Multiple Locus Variable-Number Tandem Repeats Analysis

**DOI:** 10.1186/1471-2180-6-33

**Published:** 2006-04-06

**Authors:** Florigio Lista, Giovanni Faggioni, Samina Valjevac, Andrea Ciammaruconi, Josée Vaissaire, Claudine le Doujet, Olivier Gorgé, Riccardo De Santis, Alessandra Carattoli, Alessandra Ciervo, Antonio Fasanella, Francesco Orsini, Raffaele D'Amelio, Christine Pourcel, Antonio Cassone, Gilles Vergnaud

**Affiliations:** 1 Army Medical Research Center, Via Santo Stefano Rotondo 4 00184 Rome (Italy), Via Santo Stefano Rotondo 4 00184 Rome, Italy; 2 Cattedra di Allergologia e Immunologia Clinica, II Facoltà di Medicina, Università di Roma "La Sapienza", Roma, Italy; 3 Division of Analytical Microbiology, Centre d'Etudes du Bouchet, BP3, 91710 Vert le Petit, France; 4 Institut de Génétique et Microbiologie, Université Paris Sud, 91405 Orsay Cedex, France; 5 AFSSA/LERPAZ, LNR/CNR associé Laboratoire du Charbon, 23 avenue du Général de Gaulle, 94700 Maisons-Alfort, France; 6 Istituto Superiore di Sanità, Viale Regina Elena 299 00161 Rome, Italy; 7 Istituto Zooprofilattico Sperimentale della Puglia e della Basilicata – Anthrax Reference Institute of Italy, Foggia 71100, Italy; 8 Direzione Generale della Sanità Militare, Via Santo Stefano Rotondo 4 00184 Rome, Italy

## Abstract

**Background:**

The genome of *Bacillus anthracis*, the etiological agent of anthrax, is highly monomorphic which makes differentiation between strains difficult. A Multiple Locus Variable-number tandem repeats (VNTR) Analysis (MLVA) assay based on 20 markers was previously described. It has considerable discrimination power, reproducibility, and low cost, especially since the markers proposed can be typed by agarose-gel electrophoresis. However in an emergency situation, faster genotyping and access to representative databases is necessary.

**Results:**

Genotyping of *B. anthracis *reference strains and isolates from France and Italy was done using a 25 loci MLVA assay combining 21 previously described loci and 4 new ones. DNA was amplified in 4 multiplex PCR reactions and the length of the resulting 25 amplicons was estimated by automated capillary electrophoresis. The results were reproducible and the data were consistent with other gel based methods once differences in mobility patterns were taken into account. Some alleles previously unresolved by agarose gel electrophoresis could be resolved by capillary electrophoresis, thus further increasing the assay resolution. One particular locus, Bams30, is the result of a recombination between a 27 bp tandem repeat and a 9 bp tandem repeat. The analysis of the array illustrates the evolution process of tandem repeats.

**Conclusion:**

In a crisis situation of suspected bioterrorism, standardization, speed and accuracy, together with the availability of reference typing data are important issues, as illustrated by the 2001 anthrax letters event. In this report we describe an upgrade of the previously published MLVA method for genotyping of *B. anthracis *and apply the method to the typing of French and Italian *B*. *anthracis *strain collections. The increased number of markers studied compared to reports using only 8 loci greatly improves the discrimination power of the technique. An Italian strain belonging to the B branch was described, and two new branches, D and E, are proposed. Owing to the upgrading achieved here, precise genotyping can now be produced either by automated capillary electrophoresis, or by the more accessible but slower and for some markers slightly less accurate agarose gel methodology.

## Background

*Bacillus anthracis*, a spore forming Gram positive bacteria, is the etiological agent of anthrax, a zoonosis with a worldwide distribution. The disease can be transmitted to humans by contact with infected animals or contaminated animal products. In addition to being an occupational disease, limited to farmers and veterinarians, anthrax has received considerable attention and is one of the most feared microorganism with respect to bioterrorism. In 2001, letters containing spores were mailed causing the death of five persons and several cases of cutaneous anthrax [1]. *B. anthracis *is a member of the *Bacillus cereus *group, containing *Bacillus cereus*, *Bacillus thuringiensis, Bacillus mycoides and B. anthracis *[2]. *B. anthracis *is characterized by an extremely low genetic variability, making strains differentiation very challenging [3,4]. The importance of strain differentiation of biothreat agents including *B. anthracis *is increasingly recognized as a way for identifying the source of the attack, as illustrated not only by the 2001 events but also, earlier on, by the Sverdlovsk [5] and Tokyo [6] events. Such events together suggested how crucial is the development of microbial forensics for biosecurity. Measures aimed at limiting the risk of deliberate release of dangerous pathogens, would require that isolates kept in different institutions around the world are precisely genotyped, and that the genotype profiles are shared by all countries having accepted to follow these rules, for strain accountability purposes.

Because the molecular methods classically used to differentiate between strains in other species failed to discriminate *B. anthracis *isolates, new approaches were developed. Using Amplified Fragment Length Polymorphism in 79 *B. anthracis *isolates, 31 polymorphic chromosomal regions were observed out of 1,000 fragments, and eventually, the most interesting polymorphisms turned out to be the result of tandem repeat variations [7]. Tandem repeats variability was studied most extensively in eukaryotes, and was shown to be associated with different aspects of DNA replication and recombination, including replication slippage and double-strand breaks repair [8]. They do not vary by the same mechanisms which are inducing point mutations, and the mutation rate at some Variable Number of Tandem Repeats (VNTR) loci may be considerably higher than nucleotide substitutions [9-11] in an as yet not predictable way [12,13]. The level of intraspecific polymorphism in tandemly repeated sequences varies from one locus to another and needs to be experimentally measured by typing representative strain collections. Approaches based on VNTR analysis (called MLVA for Multiple Locus VNTR Analysis) are now increasingly used to characterize in particular recently emerged, highly monomorphic pathogens including *B. anthracis *[12,14], *Mycobacterium tuberculosis *[15], *Yersinia pestis *[16]*Brucella *[17] (see [18,19] for a review). Multiple alleles can exist in the population for each tandem repeat locus. One advantage of MLVA typing as compared for instance to SNP (Single Nucleotide Polymorphism) typing is that a new isolate can be compared to previously characterised strains in an unbiased way. One weakness is that the phylogenetic value of MLVA clustering must be carefully checked and validated by using independent approaches with less homoplastic markers [20]. In previous reports, a multilocus VNTR analysis (MLVA8) method was proposed for genotyping of *B. anthracis *strains, using six chromosomal and two plasmid marker loci (*vrrA, vrrB1, vrrB2, vrrC1, vrrC2, CG3, pXO1, pXO2 *[14]). In the initial work, an automated fluorescent DNA sequencer was used to determine the size of the PCR fragments and this set of markers is now largely employed for *B. anthracis *genotyping [21-26]. However, this MLVA8 assay is not sufficient for molecular forensics approaches, the resolution achieved is too limited. For instance the vast majority of French and Italian strains have been assigned to MLVA8 genotypes (GT) 1 and 3 which differ only at one marker, located on one of the two virulence plasmids [14,22,26]. For this reason, additional markers were subsequently developed to produce an MLVA15 panel [11], and the initial clustering nomenclature [14] was revised [20]. Unfortunately none of the corresponding data has been published so far. Le Flèche *et al*. [12] extended the MLVA8 assay by proposing 14 additional markers (Bams1, 3, 5, 7, 13, 15, 21, 22, 23, 24, 25, 28, 30, 31), which greatly improved the resolution power of the assay. DNA fragment sizing was achieved on standard agarose gels stained by ethidium bromide, a technique utilising widely accessible, and very basic, equipment.

In order to have a more rapid and accurate genotyping system for *B. anthracis *we propose an automated capillary based method using essentially the panel of loci described by Le Flèche *et al *[12], with the addition of 4 new markers, and apply the method to collections of strains from Italy and France. We compare the clustering achieved with available epidemiological data for *B. anthracis*, and introduce two new *B. anthracis *groups, D and E. The present report deals with the standardization and further improvement of currently available genotyping methods for *B. anthracis*, the typing of a larger collection of strains, the comparison with available published data, and the enlargement of the publicly accessible databases.

## Results

### MLVA25 genotyping by capillary electrophoresis

**Figure 1:**
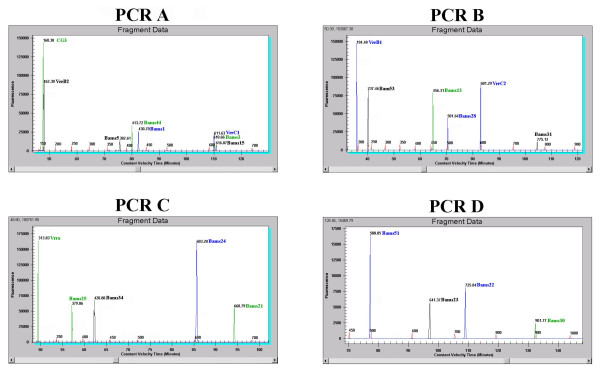
**Representative electropherograms after capillary electrophoresis separation of the fragments amplified by 4 multiplex PCR**. *B. anthracis *alleles are showed in blue, green and black peaks, whereas the red peaks are for the molecular weight size standard by Microzone.

A collection of 160 *B. anthracis *isolates, comprising strains from Italy and France and a few reference strains were analyzed to generate MLVA typing data. DNA was amplified in 4 multiplex PCR reactions, comprising 25 loci. The loci are 21 markers described previously [12,14] (Bams7 is not currently amenable to capillary electrophoresis typing because of its allele size range and was not included), and the four new markers Bams34, 44, 51, 53 (Table 1). All twenty-five markers can be amplified in only four PCR reactions by taking into account the allele size range for each locus (Table 2). The labelled amplicons were separated on a CEQ 8000 Beckmann DNA analysis system and the electropherograms were analysed by CEQ Fragment Analysis System software to determine the length of the fragments by reference to a 75–1000 bps size ladder (in red on Figure 1). After testing several primer concentrations and ratios, a balanced level of fluorescence peak intensity was obtained by using between 5 to 10 ng of template DNA per multiplex PCR reaction and the concentration of primers indicated (Table 2).

The assay was highly reproducible as tested by performing duplicate typings. The intra-experimental (within the same run) as well as the inter-experimental (between runs) variation of fragment sizing showed variations lower than one base over the whole range of PCR fragments investigated here. The accuracy of the data was determined by comparison of observed PCR fragment size to the exact values calculated by direct sequencing and available genome sequence data [27]. In some instances the amplicon size determined after electrophoresis separation did not match the expected values (Table 3). This is well illustrated for instance with Bams30 (Table 3) for which the offset increases with allele size, i.e. repeat copy number. In any case, the offset is conserved, reproducible, and the differences in calculated allele size are consistent with a 9 bp repeat unit variation.

Because Bams30 was initially identified as a 27 bp repeat unit marker [12], we investigated representative Bams30 alleles by sequencing (EMBL accession numbers AM182499 to AM182509) in order to clarify this discrepancy. Sequence data indicates that Bams30 alleles are composed of 27 base-pair units at one end of the array, and 9 bp units at the other end. Table 4A provides the allele codes of Bams30 alleles. Repeat units are not identical, different letters are used to code the different variant units which are observed [28]. Uppercase letters code for 27-base-pair repeat units whereas lowercase letters code for 9 base-pair repeat unit. The correspondence between letters and repeat units is indicated in Table 4B. The two regions are variable, and as a result the locus behaves like a 9 bp repeat unit tandem repeat.

**Figure 2:**
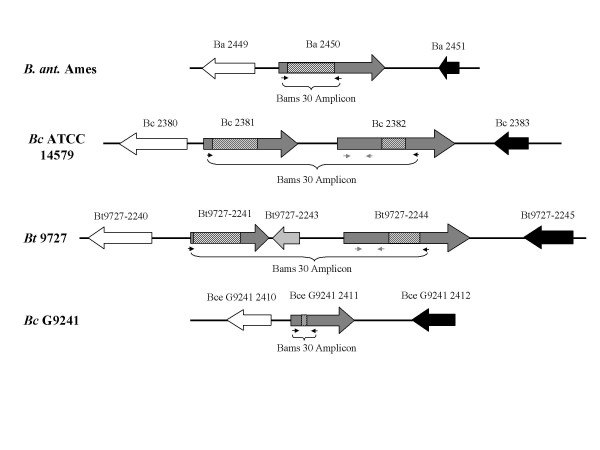
**Organization of Bams30 locus in: (B.ant. Ames), B. anthracis strain Ames; (Bc ATCC 14579), B. cereus 14579; (Bc G9241), B. cereus G9241 and B. thuringiensis 97/027**. The position of Bams30 primers is indicated by small black arrows; the primers localized in the NH2 terminus conserved part of the *Exs*H gene are indicated by small gray arrows. Tandem repeats with similarity to Bams30 are represented with striped boxes. Homologous genes are depicted using the same code.

To better understand the origin of this behaviour, we investigated the structure of this locus in the closely related *B. cereus-B. thuringiensis *group by taking advantage of available genome sequences. Figure 2 shows the organization of the locus in the genomes of *B. anthracis *(accession number AE016879), *B. cereus *ATCC14579 (AE016877), *B. cereus *G9241 [29], *B. cereus *ATCC10987 (AE017194), *B. cereus *E33L (NC_006274), and *B. thuringiensis *CEB97/027 (AE017355) also called Bt9727. The gene bearing Bams30 in *B. anthracis *(Ba2450) possess a collagen-like helix repeat and is flanked by two genes Ba2449 and Ba2451. Their homologues in the other strains were depicted using the same code. The organization in *B. cereus *G9241 is similar to *B*. *anthracis*, but in all four other strains, the situation is less simple. In *B. cereus *ATCC 14579, two genes possessing a collagen-like helix repeat protein are observed, instead of one in *B. anthracis*: Both genes possess a tandem repeat with similarity to Bams30, encoding the collagen-like repeat. Whereas in ATCC 10987, the two tandem repeats have nine base-pair repeat units, Bt9727-2241 contains a 27 bp repeat unit presumably derived from an ancestral 9 bp unit. Bt9727 is by far the closest *B. anthracis *neighbor among the 5 sequenced strains [30]. The composite organization of the Bams30 tandem repeat in *B. anthracis *can be explained by a rearrangement occurring in a Bt9727-type Bams30 locus. Strain "A1055", which is closest to the last common *B. anthracis *ancestor [20] shows the same rearrangement, which strengthens the validity of Bams30 as an interesting complementary tool for the identification of *B. anthracis *(the presence of the virulence plasmids, pXO1 and pXO2 is not sufficient since for instance "A1055" is lacking pXO1 [20]) even if the rearrangement is not strictly limited to *B. anthracis *as shown by the G9241 strain situation.

### Correspondance with agarose gel assignments

For reasons including accessibility to the different technologies, it is important to ensure that allele assignments deduced from agarose gel or capillary electrophoresis are compatible, so that the different methods can be used. For this purpose, allele sizes (expressed in basepairs, bps and converted to the number of repeat units, U) previously estimated by agarose gel were re-examined according to the capillary fragment analysis and available sequence data. For Bams3 (15 bp repeat unit) one allele, previously estimated at 609 bps (30 U) by agarose gel analysis, was corrected to 594 bps corresponding to 29 U, by capillary electrophoresis. Bams15 turned out to vary like a 9-mer rather than 18-mer repeat unit and two alleles were resolved (580 bps for 42 9-base pair units (42 U) and 598 bps for 44 U, previously grouped with 571 bps (41 U), 589 bps (43 U) or 607 bps (45 U). For Bams31 (9 bp repeat unit) one allele was resolved by capillary electrophoresis (781 bps for 65 U, previously grouped with the 772 bps for 64 U).

For markers included in the MLVA8 assay [14] which could be typed on agarose (i.e. with the exception of pXO1 and pXO2) the data obtained here were in agreement with results generated by agarose gel electrophoresis [12]. Published MLVA8 [14,21-26] data contains some allele size assignments which do not correspond to the actual size. This is due to clerical errors in the initial report [14], as noticed previously [24] and data for some loci must be converted as described in the material and methods section in order to be fully compatible.

Four new markers have been included. For Bams34, a 39-mer repeat marker, five alleles were described ranging from 238 to 581 bps (ranging from 4 to 13 repeat units). Bams44 (39-mer) and Bams53 (12-mer) show two alleles (respectively 339/417 bps or 8 and 12 repeat units, and 212/236 bps (6 and 8 repeat units)). A third allele is predicted from genome sequence data for the "Western" strain, but was not experimentally observed among the collection investigated here. For Bams51 (45-mer) four alleles were observed ranging from 338 to 538 bps (6 to 10 units).

### Data analysis

**Figure 3:**
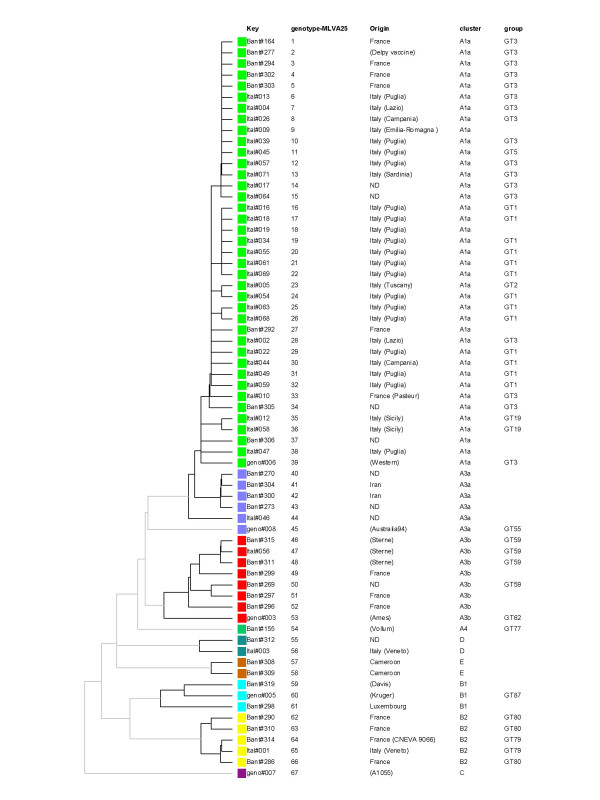
**A Clustering analysis of genotyping data**. The 67 genotypes are numbered from 1 to 67 (left-most column). The origin of the strains (when known) is indicated; ND, unknown. "Cluster" refers to the clustering and "group" refers to the genotype (GT) number based upon MLVA8 as proposed in [14]. Genotypes with no group assigned are usually minor variations of known, neighbouring groups. A number of reference strains are indicated in ().

When the eight markers comprising the MLVA8 assay are used, 30 different genotypes are resolved among the 160 isolates. With only few exceptions, these genotypes correspond to previously published profiles. Most exceptions are minor variations of previous types. The vast majority of French and Italian isolates belong to GT1 and GT3 within cluster A1a [14] in agreement with previous reports [22,26]. When the whole MLVA25 dataset is used, 67 genotypes are resolved. Isolates which are not resolved in this analysis have the same epidemiological history (when known). The clustering analysis is shown in Figure 3. Although MLVA is not by its very nature a tool with a high phylogenetic value *per se*, especially when the available strains do not fully cover the species diversity, the clustering achieved is in excellent agreement with the known relationship between the ancestral C type (genotype 67), the B cluster (genotypes 59 to 66, with the two well-separated subgroups, B1 and B2), and the A cluster (represented on one end by the "Ames" strain, initially assigned to A3b and more recently to A2 [20], and on the other end by the "Western" strain, initially assigned to A1a and more recently to A1 [20]). More precisely, the "Vollum" strain (genotype 54; strain "9" in [20]) and the "Australia94" strain (genotype 45; presumably strain "20" in [20]) are in the expected position with respect to the A group.

Genotypes 55–56, and 57–58 are loosely connected to the others, and are suggested by clustering analysis as being intermediates between the B cluster and the A cluster. This position is reminiscent of the position occupied by strains "6", "7" and "8" investigated by Pearson *et al*. [20]. It may be of interest to note that "6" which branched out in an ancestral position compared to the whole "A" cluster in the SNP analysis [20], originates from Zambia, whereas genotypes 57 and 58 in Figure 3 are from Cameroon. We propose to call these very distinct MLVA groups types D and E (Figure 3).

## Discussion

*Bacillus anthracis *genotyping is essential for epidemiological studies and for biosecurity related (including microbial forensics) issues [21]. Current methodologies such as MLVA8 are not sufficient to discriminate two geographically close isolates in natural outbreaks, since for instance most French and Italian isolates will be classified as GT1 or 3 [22,26]. Molecular characterization needs to be as precise as possible in order to discriminate isolates. The rational of this work was to update the MLVA method for quick (single-day analysis) and accurate discrimination of *Bacillus anthracis *isolates. The markers are stable, this is indirectly illustrated by the typing of "Sterne" strains from different origins. Genotypes 46 and 48 correspond to respectively two and five "Sterne" strains. Genotype 46 are the CIP (Collection Institut Pasteur) reference strains 7700 and 7702, genotype 48 covers "Sterne" vaccine strains from different countries, and the sequenced genome. The only difference in the two genotypes is for locus Bams28 [see additional file 1].

The number of VNTR loci was increased from 22 to 25 by addition of four new markers (and removal of Bams7, because allele sizes up to 2 kilobases are not adapted to typing on current capillary machines). The overall assay was converted from 25 monoplex PCR reactions, run on agarose gels and checked for size by eye or by using gel image analysis software, to 4 multiplex PCR analysed by automated capillary based fluorescent electrophoresis and internal size standard measurement for allele calculation. For most loci, accurate size estimate is achieved directly by the capillary electrophoresis machine. In other instances however, the sizing proposed by the machine, although quite reproducible, does not correspond to the real size. This could be due to the nature of the gel matrix, or to differences during migration between labelled PCR fragments and the ladder used, but most probably by a slightly biased flanking sequence or repeat unit-specific mobility pattern. Usually, the size estimate is shifted by a constant value (offset) but in some instances, the offset increases with increasing allele size (increasing repeat copy number). For this reason, allele numbering must be done carefully, using a correspondence table as presented here in Table 3. If different equipments, or reagents, are used, the correspondence indicated here must be checked by typing a number of reference strains (such as the widely available "Sterne" vaccine strain), before alleles can be confidently called and data can be merged.

Size measurement from multiplexed, automated PCR products allows for single-day isolate genotyping which is of importance in a crisis situation. It also provides a slightly increased discrimination power as compared to the MLVA20 assay run on agarose gels for the same analysed markers. This difference is essentially due to the lack of resolution of agarose for the larger, 9 bp repeat unit loci, such as Bams30 and for Bams3, Bams13, Bams15 and Bams31 typing, where new alleles were identified. For Bams15 and Bams30, capillary resolution was able to show a 9 bps repeat unit length variation instead of the respectively 18 bps and 27 unit length proposed initially.

The present investigation suggests that a recombination event at the Bams30 locus may have occurred prior to the emergence of *B. anthracis*. The event directly affects genes coding for constituents of the exosporium. Bc2381 is related to *Bcl*A, and Bc2382 corresponds to the exosporium H gene *exs*H [31]. In *B. cereus *ATCC 10987, *B. thuringiensis *97/027 (Bt9727) and *B. cereus *genome E33L, an additional ORF is located within the region. The Bams30-containing gene in *B. anthracis *is the result of a recombination between Bt9727-2241 (coding for the *Bcl*A-like protein) and Bt9727-2244 (*exs*H). The beginning and the end of the protein sequence coded by Ba2450 are respectively identical to the beginning of Bt9727-2241 and to the end of Bt9727-2244. Interestingly, a similar rearrangement is observed independently in the *B. cereus *strain G9241 which caused an anthrax like disease in a human patient [29]. As a result of this fusion, the first part of the tandem repeat itself contains a 27 bp repeat unit whereas the second part is a 9 bp repeat tandem repeat. It may be of interest to observe that two of the most polymorphic tandem repeats in *B. anthracis*, Bams13 and Bams30, are associated with genes coding for components of the exosporium (Bams13 is part of the *Bcl*A gene [32]).

## Conclusion

In this report we propose an improved automated genotyping system of *B. anthracis *by MLVA, extending the range of VNTR containing markers to 25, which increases the resolution power as compared to previous methods. The MLVA25 assay was used to genotype Italian and French isolates in a very fast and highly reproducible manner. We also better defined previously characterized alleles and describe new allelic variants. MLVA25 is a very accurate and discriminatory genotyping method and could be a promising technique for future epidemiological studies as well as differentiation of deliberated vs. naturally occurring outbreaks. The improved assay and resulting data remains compatible with the initial agarose gel based assay. The genotyping data can be queried on the web page **MLVA web-service **[33]. This site allows the comparison of local typing data to the data generated in this report.

## Methods

### Bacterial strains and isolates

The strains and DNA samples are from the collection maintained by the French Ministry of Defence at Centre d'Etude du Bouchet (CEB), from Istituto Superiore di Sanità (ISS) Italian collections and from the Italian Reference Center for Anthrax (ISZ, Foggia). Total genomic DNA was extracted as previously reported [30].

### VNTR amplification and analysis

Twenty-five loci were used, 21 from previous studies [12,14] and 4 new ones identified by comparing *B. anthracis *genome sequences using the site **GPMS, Genomes, Polymorphism and Minisatellites **[34] as described by Denoeud *et al*. [13]. Multiplex PCR reactions were prepared as follows: 5–10 ng DNA were amplified in a final volume of 15 μl containing 1× PCR Roche reaction buffer (10 mM Tris-HCl, 1,5 mM MgCl_2_, 50 mM KCl pH 8.3), 0.2 mM dNTPs (Amersham-PharmaciaTM), the appropriate concentrations of each primer (one of which was fluorescently labelled) as reported in Table 2, and 1 U Taq polymerase (Roche). The four multiplex PCR amplifications were performed on Peltier Thermal Cycler (PTC) DNA Engine DYAD or PTC200 (MJ Research) with a starting denaturation step at 96°C for 3 min, followed by 36 cycles of denaturation at 95°C for 20 seconds, annealing at 60°C for 30 seconds and extension at 65°C for 2 min. The reactions were terminated by a final incubation at 65°C for 5 min.

The PCR products were diluted 1:5 and 5 μl of the dilution was added to a mix containing 40 μl of Sample Loading Solution (SLS, Beckmann Coulter, Fullerton, CA., USA) and 0.5 μl of MicroSTEP-15a (800) size marker (Microzone Haywards Heath, UK). The samples were separated by electrophoresis in CEQ Separation Gel LPA I (Urea in buffered sieving matrix; Polyacrylamide, Beckman Coulter) on a CEQ 8000 automatic DNA Analysis System (Beckman-Coulter, Fullerton, CA., USA) with the following conditions: denaturation 90°C for 120 sec, inject 2.0 kV for 20 sec, separation 3.0 kV for 180 min. Allele sizes were estimated using CEQ Fragment Analysis System analysis software, by comparing the amplicons to the internal size standard (MicroStep-15a), consisting of fifteen labelled fragments ranging from 75 to 1000 bp.

Bams30 alleles sequencing was done as previously described [30]. The corresponding sequences accession numbers (EMBL) are AM182499-AM182509.

### MLVA data analysis

Previously published MLVA8 typing data was recovered from the literature [14,21-26]. Different conventions have been used for allele calling so that some minor corrections must be applied before merging the data. As noticed by Cheung *et al*. [24], some of the sizes initially indicated [14] do not correspond to the actual size, as can be deduced for instance from published genome sequence data. There is a 1 bp error in the *vrr*A allele sizes (for instance the allele indicated 313 in [14] is 314 bp). This has no consequences in terms of repeat unit copy number since *vrr*A has a 12 bp motif. The correction which needs to be applied to *vrr*C1 (9 bp repeat unit) is a bit more complex and as follows: the 520, 538, 583, 613 and 685 bp alleles must be converted to respectively 517, 535, 580, 616, and 688. The pXO1-aat sizes are underestimated by 3 bp. As a result the correct "Ames" strain MLVA8 code expressed in base-pairs reads 314-229-153-580-532-158-126-141 (or in terms of repeat copy numbers: 10-16-6-53-17-2-7-10) for *vrr*A, *vrr*B1, *vrr*B2, *vrr*C1, *vrr*C2, CG3, pXO1-aat and pXO2-at respectively. The "Sterne" strain is 314-229-162-580-532-158-132-135. Since at least one of these two strains is included in the cited publications, it is possible to deduce which conversion(s) needs to be applied to the corresponding data set.

The MLVA25 genotype was deduced from the genbank sequence data for strains "Ames", "Sterne", "Vollum", "Kruger", "Western", "BA1055", "Australia94".

All the data produced was managed using BioNumerics software version 4.5 (Applied Maths, Sint-Marteens-Latem, Belgium). Clustering was done using the categorical similarity coefficient and the Single Linkage Method.

## Authors' contributions

GF, ACia did the set up of the MLVA25 assay. ACia, RDes and SV participated to typing work. FL, ACas, SV, CP and GV did the error checking analysis. SV and OG did the Bams30 alleles sequence analysis. GF, ACia, ACar and ACie did various sequence analysis. RD'am and FO did error checking of overall sequence analysis. SV and CP did the Bams30 locus comparison using the different available genome sequences. GV was in charge of the Bionumerics database and clustering analyses. AF, CLD and JV maintained the Italian and French reference collections for *B. anthracis *respectively and extracted some of the DNA samples used. FL, RD'am and GV conceived the study. FL and GV wrote the report. All authors read and approved the final manuscript.

## Supplementary Material

Additional File 1 This file contains the data sets for the 67 genotypes of *B. anthracis *from Italy and France resulting in. It is a Microsoft Excel Worksheet. Click here for file

## Figures and Tables

**Table 1:** Comparison between MLVA8, MLVA20, MLVA25

**MLVA8**	**MLVA20**	**MLVA25**
pXO1		pXO1
pXO2		pXO2
*vrrA*	*vrrA*	*vrrA*
*vrrB1*	*vrrB1*	*vrrB1*
*vrrB2*	*vrrB2*	*vrrB2*
*vrrC1*	*vrrC1*	*vrrC1*
*vrrC2*	*vrrC2*	*vrrC2*
CG3	CG3	CG3
	bams1	bams1
	bams3	bams3
	bams5	bams5
	bams13	bams13
	bams15	bams15
	bams21	bams21
	bams22	bams22
	bams23	bams23
	bams24	bams24
	bams25	bams25
	bams28	bams28
	bams30	bams30
	bams31	bams31
		bams34
		bams44
		bams51
		bams53
	bams7	

**Table 2:** Composition of the 4 multiplex PCR according to the size range and the different dye label compatibility.

	Locus	Repeat units	Fragment size range (bps)	Number of alleles	New alleles	HDGI index (total n = 67)*	Primer conc. (μM)
Multiplex A	**CG3^a^**	5	153–158	2		0.50	0.30
	**bams44^a^**	39	339–417	2		0.21	0.30
	**bams3^a^**	15	474–609	7	1	0.51	0.30
	***vrrB*** **2^c^**	9	135–171	4		0.27	0.15
	**bams5^c^**	39	229–385	5	2	0.49	0.30
	**bams15^c^**	9	409–616	10	5	0.62	0.60
	**bams1^b^**	21	380–485	5		0.54	0.15
	***vrrC1* ^b^**	9	517–616	4		0.42	0.30
Multiplex B	**bams13^a^**	39	337–868	13	4	0.61	0.30
	***vrrB1* ^b^**	9	193–256	4		0.36	0,10
	**bams28^b^**	24	373–493	4	1	0.35	0.15
	***vrrC2* ^b^**	18	532–604	3		0.40	0.10
	**bams53^c^**	12	212–236	2		0.21	0.60
	**bams31^c^**	9	331–952	8	4	0.58	0.60
Multiplex C	***vrrA* ^a^**	12	290–326	4		0.40	0.06
	**bams25^a^**	15	376–391	2		0.11	0.15
	**bams21^a^**	45	541–676	3		0.09	0.30
	**bams34^c^**	39	238–581	5		0.40	0.30
	**bams24^b^**	42	469–637	6	3	0.27	0.30
Multiplex D	**bams51^b^**	45	358–538	4		0.33	0.60
	**bams22^b^**	36	555–735	5	1	0.34	0.15
	**bams23^c^**	42	399–651	5	2	0.40	0.30
	**bams30^a^**	9	268–916	12	4	0.71	0.30
	**pXO1^b^**	3	123–135	5		0.77	0.15
	**pXO2^a^**	2	132–145	8		0.58	0.15

The number of diverse allele and new allele are described for the 25 markers. Primer concentration for each set of marker in the multiplex is also reported.

* HGDI (Hunter-Gaston Diversity Index) calculated as in [36]

The alleles are identified with the number of repeats versus the former nomenclature [12]. Locus^**a**^, locus^**b**^, locus^**c **^are loci amplified by a forward primer respectively labeled with cy5.5, cy5 and WellRed dyeD2.

**Table 3:** Comparison between product sizes inferred by CEQ Fragment Analysis System software (observed) and the actual sizes obtained by sequencing data coming from direct sequencing of the PCR product (expected) or available in Genbank.

Marker	Allele fragment sizes		Alleles		**Note**
	Expected	Observed	Number of repeats	as reported [13]	
BAMS1 21 bp	380	386	11	1	
	401	407	12	2	
	422	430	13	3	
	443	451	14	4	
	485	494	16	5	
BAMS3 15 bp	474	475	21	1	
	519	520	24	2	
	549	550	26	3	
	564	565	27	4	
	579	580	28	5	new allele, previously defined as 5 or 6
	594	595	29		
	609	611	30	6	
BAMS5	229	231	3		new allele
39 bp	307	305	5	1	
	332	329	5.5		new allele
	346	343	6	2	
	385	382	7	3	
BAMS13 9 bp	337	339	17	1	
	346	348	18		new allele
	364	365	20	2	
	373	372	21		new allele
	391	393	24	3	
	427	427	27	4	
	454	456	30	5	
	463	465	31		new allele
	481	483	33		new allele
	778	785	66	7	
	814	821	70	8	
	841	849	73	9	
	868	876	76	10	
BAMS15 9 bp	409	412	23		new allele
	418	422	24	1	
	445	449	27		new allele
	535	541	37	2	
	571	579	41	3	
	580	588	42		new allele, previously defined as 3
	589	597	43	4	
	598	606	44		new allele, previously defined as 4 or 5
	607	616	45	5	
	616	627	46		new allele
BAMS21 45 bp	541	535	7	1	
	631	625	9	2	
	676	669	10	3	
BAMS22 36 bp	555	547	11	1	
	627	619	13	2	
	663	657	14		new allele
	699	690	15	3	
	735	726	16	4	
BAMS23 42 bp	399	393	5		new allele
	567	559	9		new allele
	609	600	10	1	
	639	629	10.5	2	
	651	641	11	3	
BAMS24 42 bp	469	474	8	3	
	511	519	9		new allele
	553	562	10		new allele
	582	593	10.5		new allele
	595	603	11	5	
	637	645	12	6	
BAMS25 15 bp	376	380	12	1	
	391	395	13	2	
BAMS28 24 bp	373	378	9		new allele
	397	405	10	1	
	469	478	13	2	
	493	502	14	3	
BAMS30 9 bp	268	270	6	1	
	367	369	17	2	
	493	496	31	3	
	673	681	51	4	
	691	698	53	5	
	727	735	57	6	
	754	763	60	7	
	835	848	69		new allele
	853	865	71		new allele
	862	874	72		new allele
	889	902	75	8	
	916	929	78		new allele
BAMS31 9 bp	331	332	15	1	
	637	642	49		new allele
	691	697	55		new allele
	700	705	56		new allele
	727	728	59	2	
	772	775	64	3	
	781	783	65		new allele, previously defined as 3
	952	955	84	4	
BAMS34	238	235	3		
39 bp	386	378	7		
	425	421	8		
	503	498	9		
	581	576	11		
BAMS44 39 bp	339	336	6		
	417	413	8		
BAMS51 45 bp	358	364	6		
	448	452	8		
	493	501	9		
	538	548	10		
BAMS53 12 bp	212	213	6		
	236	237	8		
vrrA 12 bp	290	289	8	1	
	302	301	9	2	
	314	314	10	3	
	326	325	11	4	
vrrB1 9 bp	193	194	12	2	
	220	221	15	3	
	229	229	16	4	
	256	256	19	5	
vrrB2 9 bp	135	137	4	1	
	153	153	6	2	
	162	162	7	3	
	171	170	8	4	
vrrC1 9 bp	517	512	46	3	
	535	530	48	4	
	583	574	53	5	
	616	611	57	6	
vrrC2 18 bp	532	528	17	1	
	568	565	19	2	
	604	601	21	3	
CG3 5 bp	153	155	1	1	
	158	160	2	2	
pXO1 3 bp	123	128	6		
	126	131	7		
	129	134	8		
	132	137	9		
	135	140	10		
pXO2 2 bp	132	136	5.5		
	133	137	6		
	135	139	7		
	137	141	8		
	139	143	9		
	141	145	10		
	143	147	11		
	145	149	12		

**Table 4:** Bams30 allele codes

Table 4A					
Genotype	cluster	Bams30 allele	PCR fragment size^a^	observed size^a^	allele coding^bc^
1	A1a	75	889	906 (+17)	**ABCD EEFGGHGGG IG GJ **abcdefcdghdghdefciefjkgk
42	A3a	75	889	906 (+17)	**ABCD EEFGGHGGG IG GJ **abcdefcdghdghdefciefjkgk
41	A3a	75	889	906 (+17)	**ABCD EEFGGHGGG IG GJ **abcdefcdghdghdefciefjkgk
33	A1a	75	889	906 (+17)	**ABCD EEFGGHGGG IG GJ **abcdefcdghdghdefciefjkgk
55	D	75	889	906 (+17)	**ABCD EEFGGHGGG IGLGJ **abcdefcdghd efciefjkgk
34	A1a	72	862	874 (+12)	**ABCD EEFGGHGGG IG J **abcdefcdghdghdefciefjkgk
37	A1a	72	862	874 (+12)	**ABCD EEFGGHGGG IG J **abcdefcdghdghdefciefjkgk
4	A1a	72	862	874 (+12)	**ABCD EEFGGHGGG IG J **abcdefcdghdghdefciefjkgk
54	A4	71	853	865 (+12)	**ABCD EEFGMHGGG I LGJ **ab cdghdghdefciefjkgk
67	C	71	853	ND	**ABCD EEFGGHGGGGGI LGJ **abcdef ciefjkgk
5	A1a	69	835	848 (+13)	**ABC EFGGHGGG IG GJ **abcdefcdghdghdefciefjkgk
58	E	60	754	763 (+9)	**ABCD EEFGG IG IJ **abcdefcdghd efciefjkgk
57	E	60	754	763 (+9)	**ABCD EEFGG IG IJ **abcdefcdghd efciefjkgk
51	A3b	57	727	735 (+8)	**ABCD EEFGGHG J **abcdefcdghd efciefjkgk
53	A3b	57	727	ND	**ABCD EEFGGHG J **abcdefcdghd efciefjkgk
52	A3b	53	691	698 (+7)	**ABCD EEFGGHG J **abcdefcdghd efjkgk
46	A3b	51	673	680 (+7)	**ABCDEEEFG J **abcdefcdghd efciefjkgk
48	A3b	51	673	681 (+7)	**ABCDEEEFG J **abcdefcdghd efciefjkgk
49	A3b	31	493	496 (+3)	**ABCD EFGGH **lj gk
63	B2	17	367	369 (+2)	**AB **cdghd efjkgk
64	B2	17	367	369 (+2)	**AB **cdghd efjkgk
66	B2	17	367	369 (+2)	**AB **cdghd efjkgk
62	B2	17	367	369 (+2)	**AB **cdghd efjkgk
59	B1	6	268	270 (+2)	**AK**
CEB9727	thuring.				**ABCDEBGIIIGGGIBJ **and ff**ff***fgdgfcafgh**abcdfabciefcfgfjka*
					
Table 4b					
27 bp repeat unit code		9 bp repeat unit code			
A	CAACAGGAATAACGGGAGCAACCGGTG	a	CAACGGGAG		
B	CAACAGGAATAACGGGAGCAACAGGTC	b	ACACAGGTC		
C	CGACGGGAACAACAGGAGCGACCGGTG	c	TAGCAGGAG		
D	CGACAGGAATAACGGGAGTGACCGGTG	d	CAACGGGCC		
E	CAACAGGAATAACGGGAGTGACCGGTG	e	CGACAGGAG		
F	CAACAGGAATAACGGGAGTGACAGGTC	f	CAACAGGTC		
G	CGACGGGAATAACGGGAGCAACAGGTC	g	CAACAGGAG		
H	CGGCGGGAATAACGGGAGTGACAGGTC	h	ATACTGGAG		
I	CGGCGGGAATAACGGGAGCAACAGGTC	i	CAACAGGCC		
J	CGACGGGAACAACAGGAGTAACCGGTC	j	TAACGGGAG		
K	CAACAGGAATAACGGGAGCAACAGGTG	k	CAACTGGTG		
L	CGACGGGAATAACGGGAGTAACCGGTC	l	CGACGGGAA		
M	CGACGGGAATAACGGGAGTAACAGGTC				

^a ^the PCR fragm ent size is the exact size, as deduced from sequence data.

The observed size is as m easured by capillary electrophoresis (the offset is indicated, and is proportional to allele size)

^b ^Upercase letters: 27 bp repeat units; Lowercase letters: 9 bp repeat units

^c ^"*" represent 9 base-pairs repeat units with a sequence m otif not observed in the *B. anthracis *alleles
